# Research on the ratio of similar materials in water-absorbent mudstone based on fuzzy mathematics

**DOI:** 10.1038/s41598-024-53409-1

**Published:** 2024-02-21

**Authors:** Chunlin Zeng, Yuejin Zhou, Xu Xiaoding

**Affiliations:** State Key Laboratory for Geomechanics and Deep Underground Engineering, China University of Mining and Technology, Xuzhou, 221116 China

**Keywords:** Civil engineering, Engineering

## Abstract

To determine the suitability and credibility of similar water-absorbent mudstone materials in model experiments, the prototype mudstone parameter similarity index was determined based on the similarity theory. Similar materials use cement and Plaster as binders and quartz sand as aggregate. The sensitivity of similar indicators of similar materials to control factors was analyzed through range statistics. Multiple regression analysis was used to establish the quantitative relationship between each control factor and similar indicators. Finally, the optimal matching scheme was refined through the combination of fuzzy mathematics and analytic hierarchy process. The results show that the physical and mechanical property indicators of similar materials with different proportions have a wide distribution range, and under certain similar conditions, they can meet the requirements of rock model tests with different properties. The aggregate-binder ratio is a direct indicator of material density, elastic modulus, and compressive strength. The main controlling factors, material density, elastic modulus, and compressive strength all increase with the decrease in aggregate-binder ratio. The cement-plaster ratio is the main control factor of material water absorption, and the water absorption gradually decreases with the increase of the cement-plaster ratio. The formula obtained through linear analysis can better represent the changing trend and distribution characteristics of various parameters of similar materials with the aggregate-binder ratio and cement-plaster ratio, and initially optimize the proportioning scheme of similar materials. Use fuzzy mathematics to evaluate the membership degree of each parameter index of similar materials, and the optimal ratio scheme was further determined to improve the credibility of later model experiments.

## Introduction

Mudstone, as a common engineering soft rock, appears frequently in surrounding rock disasters in underground engineering and is difficult to control^1–3^. Mudstone has poor structure and self-bearing capacity^4–6^. During the excavation process of mudstone tunnels, engineering disasters such as roof collapse, large deformation, and bottom drums are very prone to occur, which brings great safety risks to the construction. Mudstone is a weakly cemented rock mass^7^ with poor cementation, low strength, easy weathering, easy mud disintegration when exposed to water, and many cracks and pores. It faces the problem of difficulty in sampling. In the engineering application of mudstone, model experiments need to be conducted for actual engineering problems to predict and simulate the performance of mudstone in actual engineering^8,9^. However, due to the complexity and uncertainty of mudstone^10,11^, the results of model experiments are often affected by similar material ratios. Therefore, studying the ratio of similar mudstone materials has important practical significance and theoretical value.

In past research, scholars have achieved certain results regarding similar material ratios related to rock quality^12–15^. Based on similar model testing of lining concrete materials, Zhao^16^ used Plaster, white cement, barite powder, quartz sand, and water as raw materials and analyzed the importance of reasonable proportions of similar materials through orthogonal experiments. Xu^17^ conducted sensitivity analysis and contribution rate characterization of water-sensitive similar materials and determined the characteristics of the mechanical properties of similar materials under the influence of different factors. Fan^18^ used density as the control factor and the method of uniform formula experiment to determine the mechanical parameters of similar materials in the mine slope and analyzed the stability of the slope. Zhu^19^ simulated rock formations by using similar materials in different proportions, and then studied the formation movement characteristics of filling zone mining. Wang^20^ used river sand as the skeleton of similar materials and used additives such as nano-calcium carbonate, calcium bentonite, Plaster, and emulsified wax to create new similar materials that can simulate formation separation. Hou^21^ used the comprehensive balance method to study similar materials using sand-to-gel ratio, Plaster-talcum powder ratio, water-to-solid ratio, molding pressure, and molding time without affecting factors, providing a certain reference for similar experiments. Cui^22^ synthesized red mudstone material by means of mineral composition and ratio completely similar to natural rock samples and compared the physical and mechanical properties of synthetic red mudstone material and natural red mudstone. Kong^23^ used paraffin, quartz sand, barite powder to develop a similar material with quantitative strength reduction through temperature changes by testing the parameters of similar materials at different temperatures to simulate the strength reduction. In these scientific research works, the research on the proportion of similar rock-like materials mainly focuses on a single or a few physical and mechanical property indicators and fails to comprehensively consider the interaction between various factors. In addition, different types of materials and different factors of the same material have special properties and performance, and the reliability of experimental results cannot be guaranteed. The reliability of similar materials is related to the accuracy of experimental results. Therefore, in order to objectively reflect the mechanical phenomena in the prototype in the model experiment and maintain a certain similarity between the prototype and the model, it is necessary to find a suitable ratio of similar materials and objectively analyze and evaluate the influence of each factor. to ensure the accuracy of the experiment.

At present, mathematical models are often used in system model analysis and engineering scheme selection^24–26^. Weimin Ye build a variable weight fuzzy analytic hierarchy process, to the safety of the expansive soil side slope for evaluation^27^. Reza Mikaeil developed a dimensional stone classification system based on texture features by combining fuzzy set theory and Z-number methods^28^. Köken studied rock aggregate quality using an analytic hierarchy process, evaluating the use of four different types of rock in asphalt paving mixtures^29^. The application of fuzzy mathematics provides a theoretical basis for the digitization and quantification of fuzzy concepts, reasoning, judgment, and decision-making. The optimization of similar material schemes is a decision-making process involving multiple levels, multi-factors, multi-objectives, and multi-indicators. However, fuzzy mathematical theory cannot determine the weight of the indicator system, and the weight is mainly determined by the subjective review of experts, which has a certain subjectivity. The analytic hierarchy process can divide each factor into interrelated order levels, provide a quantitative representation of the relative importance of each level according to the judgment of objective reality, and use mathematical methods to determine the weight of the relative importance order of all elements at each level.

In this paper, the analytic hierarchy process and fuzzy mathematics theory are combined to select the matching scheme of similar materials, so as to determine the best matching scheme of similar materials. First, based on the similarity theory, the parameter similarity index of the prototype mudstone was determined, suitable binders and aggregates were selected, and the sensitivity of each similarity index of similar materials to control factors was analyzed through range statistics. Then, multiple regression analysis is used to establish the quantitative relationship between each control factor and similar indicators. Finally, the optimal ratio plan is refined through fuzzy mathematical evaluation. The research results of this article provide a more accurate and reliable similar material ratio scheme for model experiments on absorbent mudstone and improve the credibility and accuracy of model experiments. At the same time, it provides a certain reference for the optimization of the ratio of related materials.

## Similar index selection

Since mudstone is a weakly cemented soft rock, it faces the problem of difficult sampling under complex geological conditions on-site. At the same time, the occurrence of fractures is uncertain and cannot meet experimental needs. Because of this situation, domestic and foreign scholars often use similar materials of mudstone to conduct experiments when conducting related research on fractured mudstone. When using similar materials to research mudstone grouting projects, to more accurately reflect the actual situation of relevant project implementation, similar materials should meet the similarity criteria with the mudstone prototype.

Mudstone has strong water absorption and often faces deterioration when exposed to water. Therefore, the water absorption rate of mudstone is the main key indicator. In addition, when testing mudstone, its related mechanical properties affect the conduct of the experiment. Therefore, the density, stress, and Strain, elastic modulus, and compressive strength are all key indicators of similar materials. In similar simulation experiments, generally the closer to the actual proportion, the better it can reflect the actual working conditions^30^. Therefore, in the production of similar materials in this article, the similarity ratio of the key indicators is 1. According to dimensional analysis, the similarity ratio of various physical parameters of mudstone conforms to the following relationship:

1 $$C_{W} { = }C_{\varepsilon } { = }C_{\varphi } { = }C_{\mu } { = }1$$

2 $$C_{E} { = }C_{\sigma } { = }C_{{\sigma_{c} }} { = }C_{\gamma } C_{l}$$

There, CW ,Cε ,Cφ ,Cμ ,CΕ ,Cσ ,Cσc ,Cγ ,Cl are water absorption, strain, friction angle, Poisson’s ratio, elastic modulus, stress, compressive strength, weight, and geometric similarity ratio.

## Raw material selection and experimental plan

**Figure 1:**
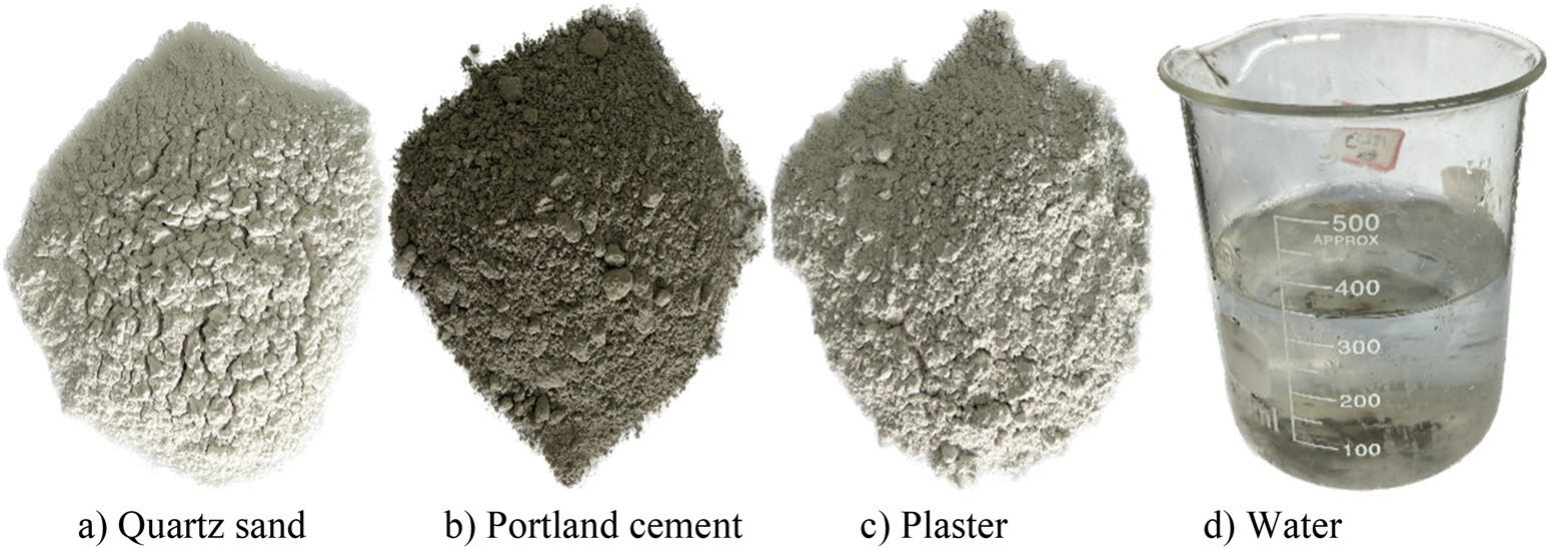
Similar material raw materials.

According to the physical characteristics of mudstone, raw materials of similar materials are selected from cement, quartz sand, Plaster, and tap water, mixed and pressed in a certain proportion. Quartz sand is used as the aggregate, with a particle size of 0.048–0.078 mm and a density of 2.71 g/cm3, which avoids the excessive roughness of the crack surface due to excessive particle size. Using cement and Plaster as binders, the strength, elastic modulus, and water absorption of similar materials can be mainly controlled by adjusting their proportions. The cement is C42.5 ordinary Portland cement produced by Shandong Yingrun Intelligent New Materials Co., Ltd., with a particle size of 0.045–0.048 mm and a density of approximately 3.10 g/cm3. The Plaster used is high-strength Plaster powder with a particle size of 0.075–0.080 mm and a density of approximately 1.54 g/cm3. The actual object is shown in Fig. 1.

When the aggregate of similar materials is quartz sand and the binder is Plaster and cement, the aggregate-binder ratio and the cement-plaster ratio are important factors in controlling the material parameters. Therefore, In order to maintain the consistency and repeatability of the experiment, the proportion of water in the total mass was set at 35%. The experiment uses the aggregate-binder ratio and the cement-plaster ratio as variables. Experimental groups were set according to different levels of aggregate-binder ratio, and each group number was set according to different levels of cement-plaster ratio to conduct the test. According to the test results of similar materials in different proportions, the optimal matching scheme of similar materials is determined. The specific experimental plan is shown in Table 1.

**Table 1:** Experimental proportioning scheme of similar materials.

Group	A	B	C	D	
aggregate-binder ratio	1:1	2:1	3:1	4:1	
Each group number	1	2	3	4	5
Cement-plaster ratio	3:1	2:1	1:1	1:2	1:3

The total quality ratio of water is 35%.

## Sample preparation and parameter testing

**Figure 2:**
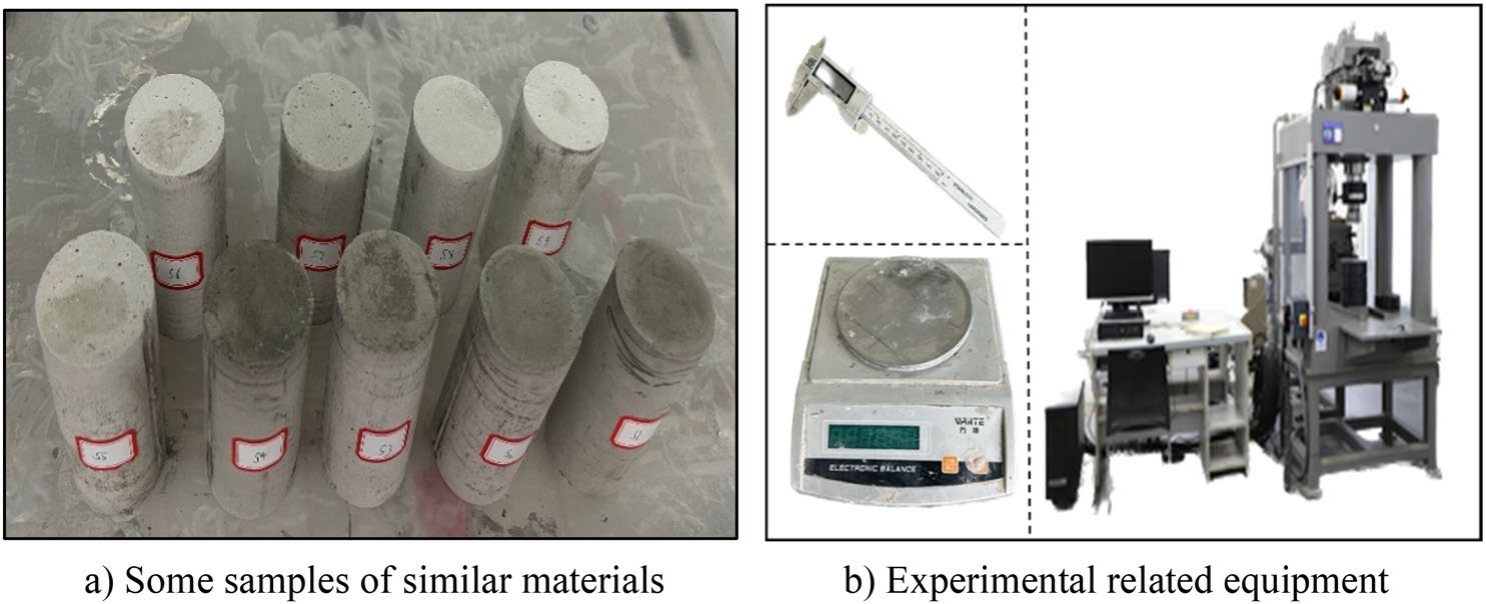
Some samples and experimental equipment of similar materials.

According to the similar material proportioning plan, weigh the required amounts of each component of each group of samples in turn, and mix them in a basin. In order to ensure the uniformity of the materials, the dry ingredients should be mixed for no less than 60 s until the dry ingredients are evenly mixed. Finally, add the measured water and continue stirring for 60 s. After stirring evenly, pour into a standard mold with a size of φ50 mm × 100 mm. At room temperature of 25 °C, let it stand for 1 day before demolding and numbering, and place the molded sample at room temperature of 25 °C for 28 days until the sample is naturally dry. Each group of samples with the same composition was prepared with 6 samples (water imbibition experiments, uniaxial tests using three samples, the experimental data based on sample testing results of the mean), totaling 120 samples. Some samples are shown in Fig. 2a. After the preparation of similar material samples is completed, the four parameters of density, water absorption, uniaxial compressive strength, and elastic modulus are experimentally measured. The production of samples and the testing of related parameters are carried out according to the standards recommended by the International Society of Rock Mechanics (ISRM). The relevant equipment is shown in Fig. 2b.

The water absorption rate W refers to the ratio of the mass of water absorbed by the rock under normal temperature and normal pressure to its mass mo before absorbing water and is often expressed as a percentage. Since mudstone is easy to disintegrate when it meets water, in order to ensure the integrity of mudstone and better reflect the water absorption performance of the material, 1 h is selected as the test time for mudstone water absorption. m_w_ is used to represent the sum of the mudstone water absorption time after 1 h. Then the water absorption rate of the mudstone in 1 h can be expressed as:

3 $$W{ = }\frac{{m_{w} - m_{o} }}{{m_{o} }} \times {\text{100\% }}$$

The density of the sample was measured by a vernier caliper and an electronic scale. It can be calculated based on the ratio of mass and volume. The compressive strength of the sample was tested on the MTS816 electro-hydraulic servo rock testing machine of China University of Mining and Technology, with a loading of 0.02 mm/s. loading rate. The elastic modulus is determined based on the full stress–strain curve obtained from uniaxial experiments. If σ1 and σ2 respectively represent the two-point stresses in the elastic stage of the full stress–strain curve, and ε1 and ε2 are the strains corresponding to the two-point stresses, then The calculation method can be expressed as:

4 $$E{ = }\frac{{\sigma_{2} - \sigma_{1} }}{{\varepsilon_{2} - \varepsilon_{1} }}$$

## Experimental results and analysis

The experimental results of density, water absorption, elastic modulus, and compressive strength of similar materials in each group are shown in Table 2. According to the test results, it can be seen that within the above range of the aggregate-binder ratio and water paste ratio, the density range of similar materials is 1.75–1.83 g/cm^3^, the water absorption range in 1 h is 3.8–5.0%, and the elastic modulus range is 0.76–1.82 GPa, and the compressive strength range is 4.213–16.622 MPa. It can be seen from the test ranges of various parameters of similar materials that, under certain similar conditions, different proportioning schemes can be used to meet similar requirements for mudstone-related experiments. In order to obtain the optimal ratio of mudstone, further analysis of the impact of various factors on similar materials is required.

**Table 2:** Test results of various groups of similar materials.

Group	Serial number	Density (g/cm^3^)	1 h water absorption rate (%)	Elastic modulus (GPa)	Compressive strength (MPa)
A	1	1.83	3.8	1.82	16.622
	2	1.81	3.9	1.76	14.546
	3	1.81	4.1	1.70	11.652
	4	1.80	4.2	1.64	10.812
	5	1.80	4.4	1.40	8.324
B	1	1.82	4.0	1.76	12.264
	2	1.81	4.1	1.56	10.583
	3	1.80	4.3	1.48	9.575
	4	1.78	4.5	1.32	7.242
	5	1.77	4.6	1.20	6.856
C	1	1.80	4.1	1.66	10.424
	2	1.79	4.3	1.52	9.988
	3	1.78	4.4	1.46	8.152
	4	1.77	4.5	1.28	7.652
	5	1.77	4.7	1.10	5.480
D	1	1.79	4.4	1.30	7.562
	2	1.79	4.4	1.13	6.542
	3	1.77	4.5	1.02	5.442
	4	1.76	4.7	1.05	5.121
	5	1.75	5.0	0.76	4.213

### Density of similar materials

**Figure 3:**
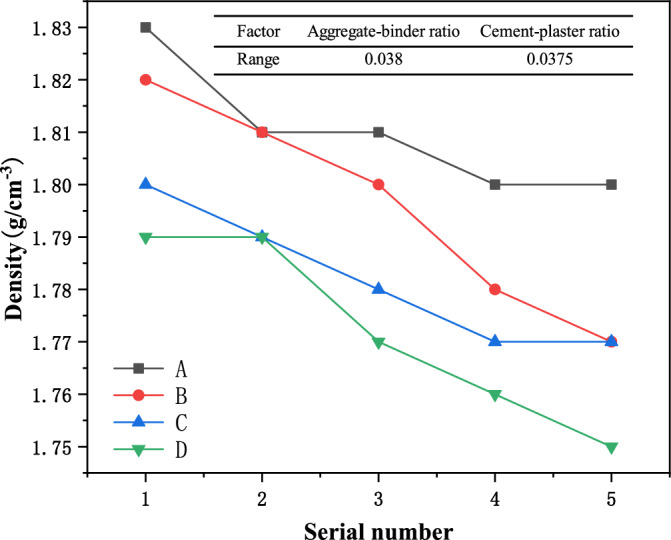
Density change curves of samples with different proportions.

The density change of the material is shown in Fig. 3. It can be seen that when the aggregate-binder ratio is constant, the density of the material gradually decreases with the decrease of the cement-plaster ratio, indicating that cement, as a cement, has good cementing ability. The higher the content, the greater the density of the specimen; when the cement-plaster ratio is constant, the density of the material generally shows a decreasing trend as the aggregate-binder ratio increases, indicating that the proportion of quartz sand can effectively control the density of the material. To analyze the influence of various factors on material density, range statistics were performed on material factors. It can be seen that the influence of the aggregate-binder ratio is more significant than that of water paste.

### Water absorption of similar materials

**Figure 4:**
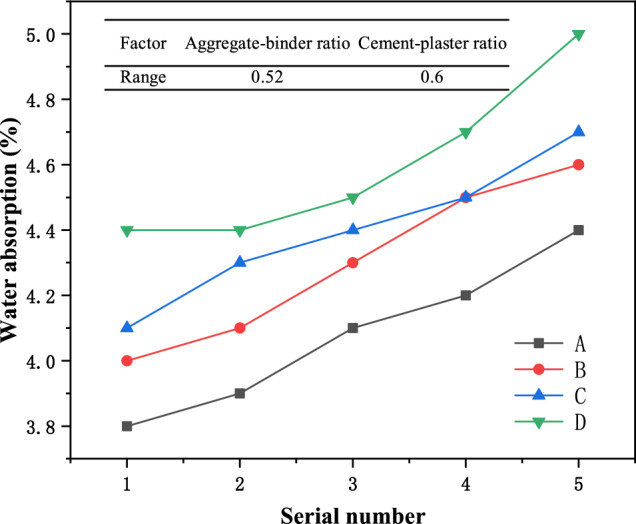
Water absorption change curves of samples with different proportions.

The change in water absorption of the material is shown in Fig. 4. It can be seen from the figure that when the aggregate-binder ratio is constant, the water absorption rate of the material gradually increases with the decrease of the cement-plaster ratio, indicating that the water absorption effect of Plaster is better than that of cement. The greater the Plaster content, the higher the water absorption rate of the specimen; when the cement-plaster ratio is constant, the water absorption rate of the material gradually increases as the aggregate-binder ratio increases. It can be seen from the results of the range statistics that for the water absorption of the material, the material is more sensitive to the cement-plaster ratio than the aggregate-binder ratio, and Plaster is the main controlling factor for the material’s water absorption.

### Elastic modulus of similar materials

**Figure 5:**
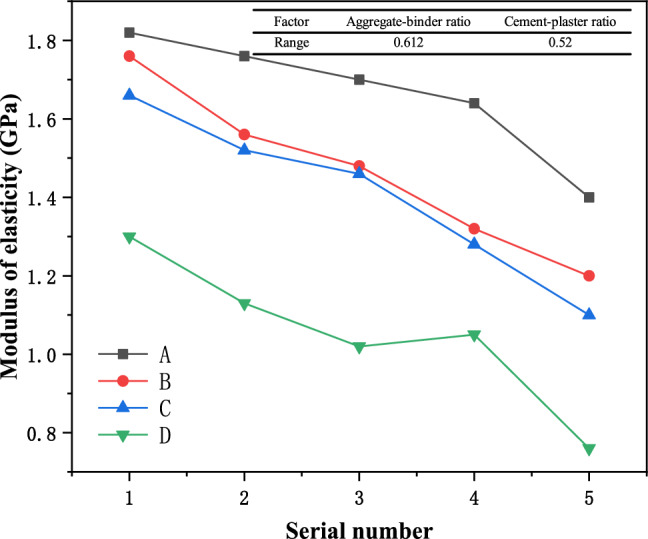
Change curves of elastic modulus of samples with different proportions.

The changes in the elastic modulus of similar materials are shown in Fig. 5. It can be seen from the figure that when the aggregate-binder ratio is constant, the elastic modulus of each group of similar materials decreases roughly linearly with the decrease in the cement-plaster ratio. It can be seen that The increase in Plaster content can enhance the deformation ability of similar materials; when the cement-plaster ratio is constant, the elastic modulus of similar materials gradually decreases with the increase in the aggregate-binder ratio. Therefore, the increase in quartz sand also provides the deformation of similar materials. space, reducing the stiffness of the material. It can be seen from the statistical results of the range of the two factors that the aggregate-binder ratio has a stronger correlation than the water paste. In the process of elastic modulus control, the influence of the aggregate-binder ratio should first be considered.

### Compressive strength of similar materials

**Figure 6:**
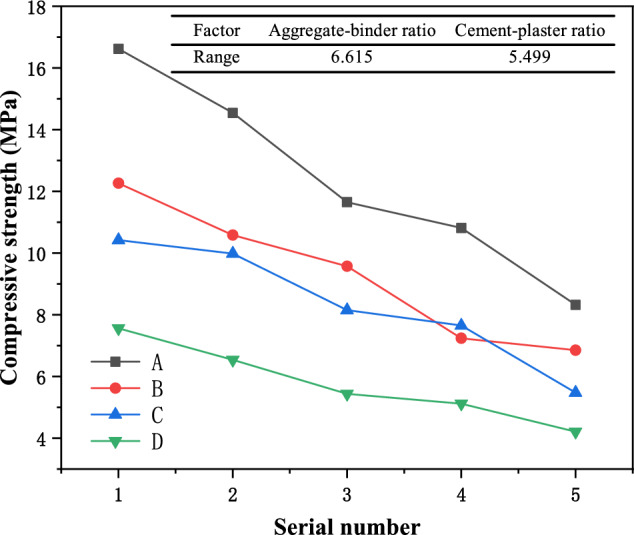
Compressive strength change curves of samples with different proportions.

The changes in uniaxial compressive strength of similar materials are shown in Fig. 6. It can be seen from the figure that the changes in compressive strength and elastic modulus of similar materials are relatively similar. When the aggregate-binder ratio is constant, the uniaxial compressive strength of similar materials in each group gradually decreases as the cement-plaster ratio decreases. It can be seen that the increase in Plaster content reduces the strength of similar materials. When the water-to-cement ratio is constant, the uniaxial compressive strength of similar materials generally shows a pattern of gradually decreasing as the aggregate-binder ratio increases. The addition of quartz sand also reduces the strength of similar materials. Through range statistical analysis, it can be seen that in terms of strength, the aggregate-binder ratio factor of similar materials is more sensitive than that of water paste.

### Quantitative correlation between parameters and influencing factors

Through the experimental results of the influencing factors of each parameter, it is found that each parameter of similar materials is jointly controlled by the two factors of aggregate-binder ratio and water paste, and their numerical correlation can be expressed by a linear relationship. Set the aggregate-binder ratio as the independent variable X1, the cement-plaster ratio as the independent variable X2; the density of similar materials as the dependent variable Y1, the water absorption rate as the dependent variable Y2, the elastic modulus as the dependent variable Y3, and the compressive strength as the dependent variable Y4. Linear regression analysis is performed based on the experimental results. R is the correlation coefficient. The results are as follows:

5 $$\left. \begin{gathered} Y_{1} = - 0.0128X_{1} + 0.013X_{2} + 1.803,{\kern 1pt} {\kern 1pt} {\kern 1pt} {\kern 1pt} {\kern 1pt} R = 0.961 \hfill \\ Y_{2} = 0.166X_{1} - 0.198X_{2} + 4.201,{\kern 1pt} {\kern 1pt} {\kern 1pt} {\kern 1pt} {\kern 1pt} {\kern 1pt} {\kern 1pt} {\kern 1pt} {\kern 1pt} {\kern 1pt} {\kern 1pt} {\kern 1pt} R = 0.946 \hfill \\ Y_{3} = - 0.189X_{1} + 0.158X_{2} + 1.653,{\kern 1pt} {\kern 1pt} {\kern 1pt} {\kern 1pt} {\kern 1pt} {\kern 1pt} {\kern 1pt} {\kern 1pt} {\kern 1pt} {\kern 1pt} R = 0.934 \hfill \\ Y_{4} = - 2.081X_{1} + 1.878X_{2} + 11.589,{\kern 1pt} {\kern 1pt} {\kern 1pt} {\kern 1pt} {\kern 1pt} R = 0.952 \hfill \\ \end{gathered} \right\}$$

**Figure 7:**
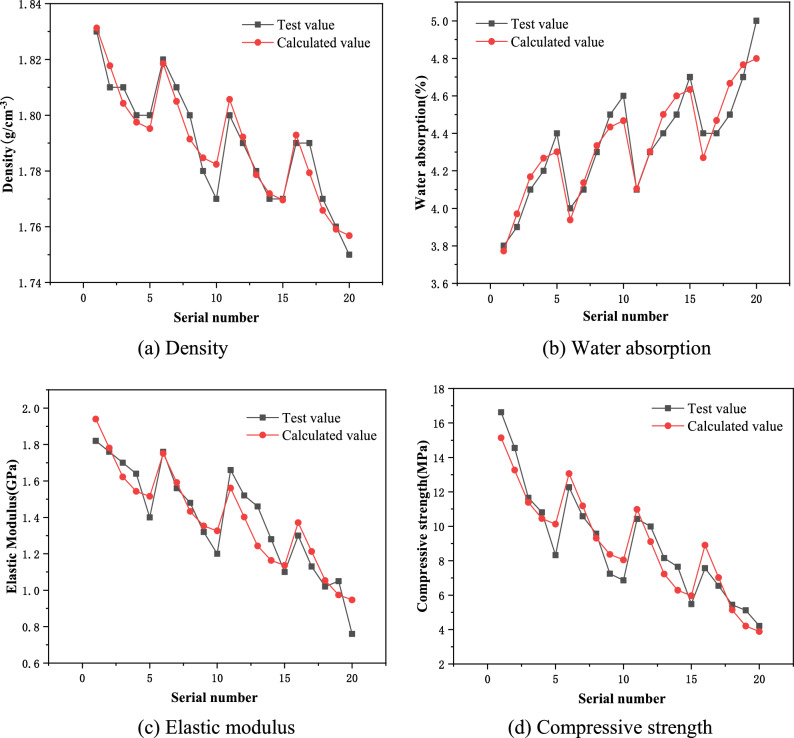
Comparison of measured values and calculated values of each parameter.

According to linear regression analysis, the comparison between the measured values and the calculated values is shown in Fig. 7. Through the comparison chart, it can be found that the formula obtained through linear analysis can better represent the changing trend and distribution characteristics of various parameters of similar materials with the aggregate-binder ratio and cement-plaster ratio. When similar materials are proportioned, each parameter can be optimized in this way to design a preliminary proportioning plan.

## Mudstone similarity evaluation

In the study of key support technologies for composite high-stress tunnels in Pingmei No. 6 Mine, a slurry-rock coupling grouting test is planned for weakly cemented mudstone to explore the slurry diffusion rules in highly water-absorbent rock formations and the stress characteristics of the bedrock on both sides of the cracks. Through on-site sampling and mechanical testing, the relevant indicators of the mudstone obtained are density 1.82 g/cm^3^, 1 h water absorption 4.3%, elastic modulus 1.61 GPa, and compressive strength 10.90 MPa. According to the above analysis, under the condition of a given similarity ratio, linear analysis can be used to pre-proportion the mudstone similar materials. Judging from the results of the proportioning plan, since there are many factors that affect similar materials in mudstone, it is difficult to make all parameters completely similar. Therefore, on the basis of linear analysis, different proportioning plans for similar materials need to be evaluated, and the preferred best mix of similar materials. The mathematical fuzzy analysis process for the experimental proportioning plan is as follows:

(1) Determination of parameter index weights. Through the basic principles of comparison scale and judgment^31,32^, the importance of comparing factors Xi and Xj is defined, and the following comparison standards can be obtained based on fuzzy mathematical analysis.

Define the comparative standard values of the elements according to Table 3. For each layer element is the reference of the parameters of the above layer, the analytic hierarchy process is used to construct the judgment matrix D for the weight of each parameter of similar materials, as follows:

$$D{ = }\left\{ {\begin{array}{*{20}l} {X_{1} /X_{1} } \hfill & {X_{1} /X_{2} } \hfill & {X_{1} /X_{3} } \hfill & {X_{1} /X_{4} } \hfill \\ {X_{2} /X_{1} } \hfill & {X_{2} /X_{2} } \hfill & {X_{2} /X_{3} } \hfill & {X_{2} /X_{4} } \hfill \\ {X_{3} /X_{1} } \hfill & {X_{3} /X_{2} } \hfill & {X_{3} /X_{3} } \hfill & {X_{3} /X_{4} } \hfill \\ {X_{4} /X_{1} } \hfill & {X_{4} /X_{2} } \hfill & {X_{4} /X_{3} } \hfill & {X_{4} /X_{4} } \hfill \\ \end{array} } \right\}{ = }\left\{ {\begin{array}{*{20}l} 1 \hfill & {1/5} \hfill & {1/3} \hfill & {1/3} \hfill \\ 5 \hfill & {{\kern 1pt} {\kern 1pt} {\kern 1pt} {\kern 1pt} {\kern 1pt} {\kern 1pt} 1} \hfill & {5/3} \hfill & {5/3} \hfill \\ 3 \hfill & {3/5} \hfill & {{\kern 1pt} {\kern 1pt} {\kern 1pt} {\kern 1pt} {\kern 1pt} {\kern 1pt} 1} \hfill & {{\kern 1pt} {\kern 1pt} {\kern 1pt} {\kern 1pt} {\kern 1pt} {\kern 1pt} 1} \hfill \\ 3 \hfill & {3/5} \hfill & {{\kern 1pt} {\kern 1pt} {\kern 1pt} {\kern 1pt} {\kern 1pt} {\kern 1pt} 1} \hfill & {{\kern 1pt} {\kern 1pt} {\kern 1pt} {\kern 1pt} {\kern 1pt} {\kern 1pt} 1} \hfill \\ \end{array} } \right\}$$

**Table 3:** Comparison standards.

Compare standard value	Definition	Compare standard value	Definition
1	Equally important	7	Strongly important
3	Slightly important	9	Extremely important
5	Obviously important	2,4,6,8	Neighbor comparison median

According to the judgment matrix, the relative weight of each parameter is calculated as follows:

6 $$w_{i} = \left( {\prod\limits_{j = 1}^{n} {Dij} } \right)^{1/n} /\sum\limits_{j = 1}^{n} {\left( {\prod\limits_{j = 1}^{n} {Dij} } \right)^{1/n} }$$

After calculation, the relative weight of each parameter of similar mudstone is W = (0.083 0.417 0.250 0.250).

(2) Determination of index membership degree. This test involves a total of 20 different similar material ratio schemes. Each scheme needs to test the density, water absorption, elastic modulus, and compressive strength of similar materials. A 4 × 20 order matrix can be formed based on similar test results. as follows:

$$A{ = }\left\{ {\begin{array}{*{20}r} \hfill {1.83} & \hfill {1.81} & \hfill {1.81} & \hfill {1.8} & \hfill {1.8} & \hfill {1.82} & \hfill {1.81} & \hfill {1.8} & \hfill {1.78} & \hfill {1.77} & \hfill {1.8} & \hfill {1.79} & \hfill {1.78} & \hfill {1.77} & \hfill {1.77} & \hfill {1.79} & \hfill {1.79} & \hfill {1.77} & \hfill {1.76} & \hfill {1.75} \\ \hfill {3.8} & \hfill {3.9} & \hfill {4.1} & \hfill {4.2} & \hfill {4.4} & \hfill 4 & \hfill {4.1} & \hfill {4.3} & \hfill {4.5} & \hfill {4.6} & \hfill {4.1} & \hfill {4.3} & \hfill {4.4} & \hfill {4.5} & \hfill {4.7} & \hfill {4.4} & \hfill {4.4} & \hfill {4.5} & \hfill {4.7} & \hfill 5 \\ \hfill {1.82} & \hfill {1.76} & \hfill {1.7} & \hfill {1.64} & \hfill {1.4} & \hfill {1.76} & \hfill {1.56} & \hfill {1.48} & \hfill {1.32} & \hfill {1.2} & \hfill {1.66} & \hfill {1.52} & \hfill {1.46} & \hfill {1.28} & \hfill {1.1} & \hfill {1.3} & \hfill {1.13} & \hfill {1.02} & \hfill {1.05} & \hfill {0.76} \\ \hfill {16.62} & \hfill {14.54} & \hfill {11.65} & \hfill {10.81} & \hfill {8.32} & \hfill {12.26} & \hfill {10.58} & \hfill {9.57} & \hfill {7.24} & \hfill {6.85} & \hfill {10.42} & \hfill {9.98} & \hfill {8.15} & \hfill {7.65} & \hfill {5.48} & \hfill {7.56} & \hfill {6.54} & \hfill {5.44} & \hfill {5.12} & \hfill {4.21} \\ \end{array} } \right\}$$

The membership degree of the quantitative index is determined by the membership function method, with K = {k_i1_, k_i2_,… , k_ij_} denotes the membership degree of similar mudstone materials, k_ij_ denotes the membership degree of the i-th index of similar material of the j-th ratio scheme, A = {a_i1_, a_i2_,… a_ij_} represents the test result of similar materials, a_i_ represents the i-th index value of mudstone, a_ij_ represents the i-th index value of similar materials of the j-th ratio scheme, and c_i_ represents the i-th index similarity ratio of mudstone. The membership degree of similar material index can be calculated by Formula 7, and the higher the value, the higher the fit degree with the original rock parameter index.

7 $$k_{ij} = 1 - \left| {\frac{{a_{i} - c_{i} a_{ij} }}{{a_{i} }}} \right|$$

Substituting the original rock parameters and test results, we can get:

$$\begin{gathered} K{ = }\left\{ {\begin{array}{*{20}r} \hfill {0.995} & \hfill {0.995} & \hfill {0.995} & \hfill {0.99} & \hfill {0.99} & \hfill 1 & \hfill {0.995} & \hfill {0.99} & \hfill {0.979} & \hfill {0.973} \\ \hfill {0.884} & \hfill {0.907} & \hfill {0.954} & \hfill {0.977} & \hfill {0.977} & \hfill {0.931} & \hfill {0.954} & \hfill 1 & \hfill {0.954} & \hfill {0.931} \\ \hfill {0.87} & \hfill {0.907} & \hfill {0.945} & \hfill {0.982} & \hfill {0.87} & \hfill {0.907} & \hfill {0.969} & \hfill {0.92} & \hfill {0.82} & \hfill {0.746} \\ \hfill {0.476} & \hfill {0.666} & \hfill {0.932} & \hfill {0.992} & \hfill {0.764} & \hfill {0.875} & \hfill {0.971} & \hfill {0.879} & \hfill {0.665} & \hfill {0.629} \\ \end{array} } \right. \hfill \\ \left. {{\kern 1pt} {\kern 1pt} {\kern 1pt} {\kern 1pt} {\kern 1pt} {\kern 1pt} {\kern 1pt} {\kern 1pt} {\kern 1pt} {\kern 1pt} {\kern 1pt} {\kern 1pt} {\kern 1pt} {\kern 1pt} {\kern 1pt} {\kern 1pt} {\kern 1pt} {\kern 1pt} {\kern 1pt} {\kern 1pt} \begin{array}{*{20}r} \hfill {0.99} & \hfill {0.984} & \hfill {0.979} & \hfill {0.973} & \hfill {0.973} & \hfill {0.984} & \hfill {0.984} & \hfill {0.973} & \hfill {0.968} & \hfill {0.962} \\ \hfill {0.954} & \hfill 1 & \hfill {0.977} & \hfill {0.954} & \hfill {0.907} & \hfill {0.976} & \hfill {0.977} & \hfill {0.954} & \hfill {0.907} & \hfill {0.838} \\ \hfill {0.969} & \hfill {0.945} & \hfill {0.907} & \hfill {0.796} & \hfill {0.684} & \hfill {0.808} & \hfill {0.702} & \hfill {0.634} & \hfill {0.653} & \hfill {0.473} \\ \hfill {0.957} & \hfill {0.917} & \hfill {0.748} & \hfill {0.703} & \hfill {0.503} & \hfill {0.694} & \hfill {0.601} & \hfill {0.5} & \hfill {0.47} & \hfill {0.387} \\ \end{array} } \right\} \hfill \\ \end{gathered}$$

Based on the relative weight and membership degree of each parameter, the fuzzy linear weighted transformation method is used to comprehensively evaluate the overall membership degree. The calculation method is as follows:

8 $$B = W*K$$

After calculation, the comprehensive evaluation value of the overall membership degree of different schemes for similar mudstones is B = (0.787 0.854 0.949 0.983 0.898 0.916 0.965 0.948 0.850 0.812 0.961 0.964 0.902 0.853 0.755 0.864 0.8 14 0.762 0.739 0.644), the total membership value of different schemes represents It refers to the degree of compatibility with similar mudstone. The larger the value, the better the similarity with the mudstone. After comparison, it can be seen that the fourth proportioning plan of similar mudstone is the best proportioning plan for similar materials.

## Conclusion

(1) Through the test of related indicators of similar water-absorbent mudstone materials, the density range of similar materials is 1.75–1.83 g/cm^3^, the water absorption rate range of 1 h is 3.8–5.0%, the elastic modulus range is 0.76–1.82 GPa, and the compressive strength The range is 4.213–16.622 MPa. Under certain similar conditions, different proportioning schemes can be used to meet similar requirements for mudstone-related experiments.

(2) Through the sensitivity analysis of each factor, it can be seen that the aggregate-binder ratio is the main controlling factor of material density, elastic modulus, and compressive strength. The material density, elastic modulus, and compressive strength all increase as the aggregate-binder ratio decreases. Decreases with the decrease of the cement-plaster ratio; the cement-plaster ratio is the main control factor of the material’s water absorption. The material’s water absorption gradually decreases with the increase of the cement-plaster ratio, and gradually increases with the increase of the aggregate-binder ratio; adjust each factor The specific gravity can control the mechanical properties of similar materials;

(3) The formula obtained through linear analysis can better represent the changing trends and distribution characteristics of various parameters of similar materials with the aggregate-binder ratio and cement-plaster ratio. When similar materials are proportioned, each parameter can be optimized in this way to design a preliminary proportioning plan;

(4) The evaluation method of fuzzy mathematical analysis can be used to further determine the optimal ratio of similar materials. Based on the analysis of on-site mudstone samples in a mine in Henan, through the study of each membership degree of different schemes, it can be seen that the optimal ratio of similar materials for water-absorbent mudstone The proportioning plan is quartz sand: cement: Plaster = 3:1:2. The optimal proportioning plan is refined through fuzzy mathematical evaluation.

## Data Availability

The data presented in this study are available on request from the corresponding author.
